# Motor Vehicle Crash Risk in Older Adult Drivers With Attention-Deficit/Hyperactivity Disorder

**DOI:** 10.1001/jamanetworkopen.2023.36960

**Published:** 2023-10-04

**Authors:** Yuxin Liu, Stanford Chihuri, Thelma J. Mielenz, Howard F. Andrews, Marian E. Betz, Carolyn DiGuiseppi, David W. Eby, Linda L. Hill, Vanya Jones, Lisa J. Molnar, David Strogatz, Guohua Li

**Affiliations:** 1 Department of Epidemiology, Mailman School of Public Health, Columbia University, New York, New York; 2 Department of Anesthesiology, Vagelos College of Physicians and Surgeons, Columbia University, New York, New York; 3 Center for Injury Science and Prevention, Columbia University Irving Medical Center, New York, New York; 4 Department of Psychiatry, Vagelos College of Physicians and Surgeons, Columbia University, New York, New York; 5 Department of Biostatistics, Mailman School of Public Health, Columbia University, New York, New York; 6 Department of Emergency Medicine, University of Colorado School of Medicine, Aurora; 7 VA Eastern Colorado Geriatric Research Education and Clinical Center, Aurora; 8 Department of Epidemiology, Colorado School of Public Health, University of Colorado Anschutz Medical Campus, Aurora; 9 University of Michigan Transportation Research Institute, Ann Arbor; 10 School of Public Health, University of California, San Diego, La Jolla; 11 Department of Health, Behavior and Society, Johns Hopkins Bloomberg School of Public Health, Baltimore, Maryland; 12 Bassett Research Institute, Cooperstown, New York

## Abstract

**Importance:**

Symptoms of attention-deficit/hyperactivity disorder (ADHD), such as inattentiveness and impulsivity, could affect daily functioning and driving performance throughout the life span. Previous research on ADHD and driving safety is largely limited to adolescents and young adults.

**Objective:**

To examine the prevalence of ADHD and the association between ADHD and crash risk among older adult drivers.

**Design, Setting, and Participants:**

This prospective cohort study collected data from primary care clinics and residential communities in 5 US sites (Ann Arbor, Michigan; Baltimore, Maryland; Cooperstown, New York; Denver, Colorado; and San Diego, California) between July 6, 2015, and March 31, 2019. Participants were active drivers aged 65 to 79 years at baseline enrolled in the Longitudinal Research on Aging Drivers project who were studied for up to 44 months through in-vehicle data recording devices and annual assessments. The data analysis was performed between July 15, 2022, and August 14, 2023.

**Exposure:**

Lifetime ADHD based on an affirmative response to the question of whether the participant had ever had ADHD or had ever been told by a physician or other health professional that he or she had ADHD.

**Main Outcomes and Measures:**

The main outcomes were hard-braking events defined as maneuvers with deceleration rates of 0.4*g* or greater, self-reported traffic ticket events, and self-reported vehicular crashes. Multivariable negative binomial modeling was used to estimate adjusted incidence rate ratios (aIRRs) and 95% CIs of outcomes according to exposure status.

**Results:**

Of the 2832 drivers studied, 1500 (53.0%) were women and 1332 (47.0%) were men with a mean (SD) age of 71 (4) years. The lifetime prevalence of ADHD in the study sample was 2.6%. Older adult drivers with ADHD had significantly higher incidence rates of hard-braking events per 1000 miles than those without ADHD (1.35 [95% CI, 1.30-1.41] vs 1.15 [95% CI, 1.14-1.16]), as well as self-reported traffic ticket events per 1 million miles (22.47 [95% CI, 16.06-31.45] vs 9.74 [95% CI, 8.99-10.55]) and self-reported vehicular crashes per 1 million miles (27.10 [95% CI, 19.95-36.80] vs 13.50 [95% CI, 12.61-14.46]). With adjustment for baseline characteristics, ADHD was associated with a significant 7% increased risk of hard-braking events (aIRR, 1.07; 95% CI, 1.02-1.12), a 102% increased risk of self-reported traffic ticket events (aIRR, 2.02; 95% CI, 1.42-2.88), and a 74% increased risk of self-reported vehicular crashes (aIRR, 1.74; 95% CI, 1.26-2.40).

**Conclusions and Relevance:**

As observed in this prospective cohort study, older adult drivers with ADHD may be at a significantly elevated crash risk compared with their counterparts without ADHD. These findings suggest that effective interventions to improve the diagnosis and clinical management of ADHD among older adults are warranted to promote safe mobility and healthy aging.

## Key Points

**Question:**

Is crash risk associated with attention-deficit/hyperactivity disorder (ADHD) in older adult drivers?

**Findings:**

In this cohort study of 2832 active drivers aged 65 to 79 years, the lifetime prevalence of ADHD was 2.6%. Having ADHD was associated with a 7% increased risk of hard-braking events, a 102% increased risk of self-reported traffic ticket events, and a 74% increased risk of self-reported vehicular crashes.

**Meaning:**

Given this finding that ADHD is associated with a significantly increased crash risk in older adult drivers, improved diagnosis and clinical management of ADHD to promote safe mobility and healthy aging are warranted.

## Teaser

This cohort study examines the prevalence of attention-deficit/hyperactivity disorder in older adult drivers and its association with vehicular crash risk.

## Introduction

Attention-deficit/hyperactivity disorder (ADHD) is a chronic neurodevelopmental condition with symptoms such as inattentiveness, impulsivity, and hyperactivity.^1^ Although ADHD is commonly considered a childhood disorder, it can persist into adulthood and affect daily life performances of older adults.^2,3^ In the US, the reported prevalence of ADHD is 9.8% to 12.9% in children younger than 17 years and 8.1% in adults aged 18 to 44 years.^4,5,6^

The reported prevalence of ADHD in adults has increased in recent years due to improved diagnosis. Data from the National Ambulatory Medical Care Survey indicated a 2-fold increase in the diagnosis of ADHD in adults compared with youth from 2008-2009 to 2012-2013.^7^ Similarly, a longitudinal study found a 4-fold increase in the diagnosis of ADHD in adults compared with children from 2007 to 2016.^8^ Although concerns remain about contributing factors for the reported increase in adult cases of ADHD, there is mounting evidence that the prevalence of ADHD in adults has increased in recent years.^9^ In general, the prevalence of ADHD decreases with advancing age, as only approximately 30% of children with ADHD have persistent symptoms throughout their life span.^10^ The age-related decline in symptoms coupled with the presence of psychiatric comorbidities, such as anxiety, depression, and bipolar disorder, could impede the diagnosis and treatment of ADHD in adults.^10,11,12^

Major symptoms of ADHD may impair cognitive functioning, including complex executive functioning in activities of daily living, such as driving. Previous research showed a positive association between ADHD and unsafe driving behaviors based on self-, informant, and official reports.^13^ For on-road driving outcomes, drivers with ADHD were more prone to receive license suspensions and speeding tickets and to be involved in vehicular crashes.^14^ A meta-analysis found that having ADHD was associated with an 88% increased risk in self-reported motor vehicle crashes.^15^ Experimental studies using driving simulators revealed that participants with ADHD had higher average speed, more frequent lane changes, worse vehicle control, a greater likelihood of collisions with an obstacle, and a higher propensity of other adverse driving outcomes than participants without ADHD.^16,17^ Risk factors, such as heightened anxiety, could contribute to adverse driving outcomes, such as traffic citations, of individuals with ADHD.^18^ Moreover, potentially inappropriate medication use is associated with unsafe driving behaviors, as older drivers take more medications than younger drivers.^19^ However, it remains unclear whether the association of polypharmacy use with crash risk is similar between drivers with and without ADHD.

The proportion of the US licensed driver population accounted for by older adults (ie, those aged 65 years or older) increased from 13% in 2011 to 17% in 2020.^20^ It is well established that older adult drivers have substantially higher fatal crash rates per mile driven than drivers aged 30 to 59 years.^21^ Despite the aging US driver population and the increasing prevalence of ADHD in adults, research on ADHD and driving safety is limited primarily to adolescents and young adults.^17,18^ Few studies have assessed the association of ADHD with crash risk among older adults. In this study, we aimed to examine the prevalence of ADHD and test the hypothesis that ADHD is associated with a significantly increased motor vehicle crash risk in older adult drivers.

## Methods

### Study Sample

The Longitudinal Research on Aging Drivers (LongROAD) project was a multisite, prospective cohort study of 2990 active drivers aged 65 to 79 years at baseline. The sample was recruited between July 6, 2015, and March 31, 2017 from primary care clinics or health care systems in 5 sites (Ann Arbor, Michigan; Baltimore, Maryland; Cooperstown, New York; Denver, Colorado; and San Diego, California).^21^ Each site recruited approximately the same number of study participants.^21^ Driving data, such as hard-braking events and total miles driven, were collected for up to 44 months through an in-vehicle data recording device (DataLogger; Danlaw, Inc) installed to an accessible on-board diagnostics II port in the participant’s primary vehicle at the time of enrollment.

The eligibility criteria for participating in the LongROAD project were as follows: aged 65 to 79 years at baseline with a valid driver’s license, driving at least once a week on average, residing in the study site catchment area for at least 10 months a year, having no plans to move outside of the catchment area within 5 years, having access to a motor vehicle of the model year 1996 or newer with an accessible on-board diagnostics II port, driving 1 vehicle approximately 80% of the time or more if having access to more than 1 vehicle, being fluent in English, and scoring 4 or higher on the Six-Item Screener.^21^ Older adults with significant cognitive impairment or Alzheimer disease and related dementias were not eligible for enrollment.^21^ Excluded from this study were 138 participants due to missing data on ADHD status, 19 participants due to missing driving data, and 1 participant due to unreliable data on hard-braking events, yielding 2832 participants for analysis.

An annual questionnaire consisting of driving, health, and functioning domains was used to collect self-reported data, such as demographic characteristics, driving performance, and health behaviors. The brown-bag review method was used to collect data on medications and supplements, which were coded according to the pharmacologic and therapeutic classification system established by the American Society of Health-System Pharmacists in the American Hospital Formulary Service Clinical Drug Information.^22^

Informed consent was obtained from participants, and a certificate of confidentiality was obtained from the National Institutes of Health. The study protocol was approved by the institutional review boards of all involved institutions. The design and methods of the LongROAD project are described in detail elsewhere.^21^ While preparing this article, we followed the Strengthening the Reporting of Observational Studies in Epidemiology (STROBE) reporting guideline.

### ADHD

The exposure of interest was self-reported lifetime ADHD recorded at baseline. As part of the health and behavior assessment, participants were asked if they have ever had or ever been told by a physician or other health professional that they had “attention-deficit disorder/ADD” or “attention-deficit/hyperactivity disorder/ADHD.” An affirmative answer to the question was coded as having ADHD. Data on attention-deficit disorder and ADHD were combined in this study because attention-deficit disorder is no longer a separate diagnosis according to the *Diagnostic and Statistical Manual of Mental Disorders* (Fifth Edition).^23^ Self-reported lifetime diagnoses of depression and anxiety were collected at baseline.

### Outcomes of Interest

The outcome of primary interest was hard-braking events, which were defined as maneuvers with deceleration rates of 0.4*g* or higher. Secondary outcomes included self-reported traffic violations and crashes collected during the annual follow-up assessment. Data for self-reported traffic ticket events were collected from answers to the question, “How many times in the past year have you received a traffic ticket, other than a parking ticket, where you were found to be guilty, regardless of whether you think you were at fault?” Data for self-reported crashes were collected from answers to the question, “How many accidents have you been involved in over the past year when you were the driver?” All data related to the 3 outcomes of interest were collected during the first 3 years of follow-up (ending on March 31, 2019). The response rates during the follow-up were 96.6% in the first year, 94.1% in the second year, and 93.9% in the third year.

### Covariates

Demographic and health characteristics were obtained through in-person visits at the study sites. Self-reported data related to age (categorized as 65-69 years, 70-74 years, and 75-79 years), sex, race and ethnicity (categorized as non-Hispanic Black, non-Hispanic White, and other), marital status, highest degree or level of education, annual household income in total, and urbanicity (classified by Rural-Urban Commuting Area Code) were collected during baseline visits. Self-reported race and ethnicity were recorded because fatality rates from motor vehicle crashes vary considerably across racial and ethnic groups.^24^ The other racial and ethnic group included small numbers of Asian, Native and Indigenous American, and Hispanic participants.

### Statistical Analysis

Statistical analysis was performed between July 15, 2022, and August 14, 2023. The sample size of the LongROAD project was estimated to generate adequate person-time of observation for ensuring a study power of more than 80% in detecting a crash incidence rate ratio (IRR) of 3.0 or greater.^21^ The post hoc powers for detecting the differences at the *P* = .05 level between drivers with and without ADHD in the incidence rates of hard-braking events, traffic ticket events, and vehicular crashes per mile driven were 100%, 97%, and 96%, respectively. Pearson χ^2^ tests were used to examine differences in the prevalence of ADHD across covariates. Incidence rates of hard-braking events per 1000 miles driven and self-reported traffic ticket events and vehicular crashes per 1 million miles driven were computed according to baseline demographic characteristics, number of medications the participant was taking, comorbidities (anxiety and depression), and ADHD. Crude IRRs for hard-braking events were computed by dividing the total number of hard-braking events by the total miles driven in each stratum of participant characteristics. Due to overdispersion, multivariable negative binomial modeling was used to estimate adjusted IRRs (aIRRs) and 95% CIs of the 3 outcomes, with miles driven as the offset. A 2-tailed *P* < .05 was considered statistically significant. For parsimoniousness of the models, only statistically significant covariates were included. All data analyses were performed using SAS OnDemand for Academics, version 3.81 software (SAS Institute Inc).

## Results

Of the 2832 older adult drivers included in this study, 1179 (41.6%) were aged 65 to 69 years at baseline, 984 (34.8%) were aged 70 to 74 years, and 669 (23.6%) were aged 75 to 79 years (mean [SD] age, 71 [4] years). More than one-half (1500 [53.0%]) of the study participants were women (vs 1332 men [47.0%]), 2423 (85.7%) were non-Hispanic White (vs 211 non-Hispanic Black [7.5%] and 194 other race and ethnicity [6.8%]), 1774 (63.2%) were currently married, 1807 (64.0%) had a bachelor’s or graduate degree, 1988 (72.8%) had an annual household income of $50 000 or greater, 2052 (72.5%) lived in urban areas, and 879 (31.5%) were taking 10 or more medications (Table 1). The lifetime prevalence of self-reported anxiety and depression were 11.3% (318 participants) and 19.8% (560 participants), respectively.

**Table 1.** Incidence Rates of Hard-Braking Events by Baseline Demographic and Health Characteristics, the Longitudinal Research on Aging Drivers Project

Variable	No. (%) of drivers (N = 2832)^a^	Total miles	Total hard-braking events	Incidence rate per 1000 miles (95% CI)
Age, y				
65-69	1179 (41.6)	28 252 613	30 628	1.08 (1.07-1.10)
70-74	984 (34.8)	22 312 387	26 082	1.17 (1.15-1.18)
75-79	669 (23.6)	12 997 752	16 807	1.29 (1.27-1.31)
Sex				
Male	1332 (47.0)	32 523 913	37 070	1.14 (1.13-1.15)
Female	1500 (53.0)	31 038 839	36 447	1.17 (1.16-1.19)
Race and ethnicity				
Black, non-Hispanic	211 (7.5)	4 555 643	6001	1.32 (1.28-1.35)
White, non-Hispanic	2423 (85.7)	55 197 063	61 029	1.11 (1.10-1.11)
Other^b^	194 (6.8)	3 738 595	6332	1.69 (1.65-1.74)
Marital status				
Married	1774 (63.2)	41 906 818	45 086	1.08 (1.07-1.09)
Not married	1032 (36.8)	21 136 355	27 678	1.31 (1.29-1.33)
Education				
High school or less	318 (11.3)	6 397 675	6903	1.08 (1.05-1.10)
Associate’s degree	698 (24.7)	15 979 680	19 949	1.25 (1.23-1.27)
Bachelor’s degree	662 (23.4)	14 670 741	17 128	1.17 (1.15-1.19)
Advanced degree	1145 (40.6)	26 306 535	29 348	1.12 (1.10-1.13)
Annual household income, $				
≤49 999	743 (27.2)	14 893 360	19 213	1.29 (1.27-1.31)
50 000-99 999	1095 (40.1)	26 173 915	27 208	1.04 (1.03-1.05)
≥100 000	893 (32.7)	20 412 181	24 876	1.22 (1.20-1.23)
Urbanicity				
Urban	2052 (72.5)	42 080 430	58 661	1.39 (1.38-1.41)
Suburban or rural	780 (27.5)	21 482 322	14 856	0.69 (0.68-0.70)
ADHD				
Yes	75 (2.6)	1 530 011	2071	1.35 (1.30-1.41)
No	2757 (97.4)	62 032 741	71 446	1.15 (1.14-1.16)
Anxiety				
Yes	318 (11.3)	6 216 559	7812	1.26 (1.23-1.28)
No	2504 (88.7)	57 202 356	65 585	1.15 (1.14-1.16)
Depression				
Yes	560 (19.8)	11 039 249	14 384	1.30 (1.28-1.32)
No	2269 (80.2)	52 471 581	59 108	1.13 (1.12-1.14)
No. of medications				
0-4	801 (28.7)	19 126 437	20 466	1.07 (1.06-1.08)
5-9	1111 (39.8)	25 508 121	28 109	1.10 (1.09-1.11)
≥10	879 (31.5)	18 217 109	24 043	1.32 (1.30-1.34)

Abbreviation: ADHD, attention-deficit/hyperactivity disorder.

^a^ The total number of drivers by different covariates may vary due to exclusion of missing data.

^b^ Other race and ethnicity includes Asian, Native and Indigenous American, and Hispanic participants.

Of the 75 drivers who reported having ADHD at baseline, 57 had attention deficit disorder, 29 had ADHD, and 11 had both, yielding an overall lifetime prevalence of 2.6% (Table 1). The prevalence of ADHD differed significantly between those taking 10 or more medications and those taking fewer than 10 medications (4.8% vs 1.5%; *P* < .001), between those with and without anxiety (7.2% vs 2.0%; *P* < .001), and between those with and without depression (7.3% vs 1.4%; *P* < .001). The prevalence of ADHD was not significantly associated with age, sex, race and ethnicity, marital status, education, annual household income, and urbanicity.

The incidence rates of hard-braking events per 1000 miles were 1.35 (95% CI, 1.30-1.41) for older adult drivers with ADHD and 1.15 (95% CI, 1.14-1.16) for those without ADHD. Significantly increased incidence rates of hard-braking events were also found among those who had anxiety (1.26; 95% CI, 1.23-1.28) or depression (1.30; 95% CI, 1.28-1.32); were aged 75 to 79 years (1.29; 95% CI, 1.27-1.31), female (1.17; 95% CI, 1.16-1.19), non-Hispanic Black (1.32; 95% CI, 1.28-1.35), or other race and ethnicity (1.69; 95% CI, 1.65-1.74); were not married (1.31; 95% CI, 1.29-1.33); lived in urban areas (1.39; 95% CI, 1.38-1.41); or were taking 10 or more medications (1.32; 95% CI, 1.30-1.34) (Table 1).

The overall incidence rates per 1 million miles of self-reported traffic ticket events and vehicular crashes were 10.04 (95% CI, 9.29-10.86) and 13.83 (95% CI, 12.94-14.78), respectively. These outcomes varied markedly with baseline demographic and health characteristics (Table 2). Older adult drivers with ADHD were more than twice as likely as their counterparts without ADHD to report being involved in traffic ticket events (incidence rate per 1 million miles, 22.47 [95% CI, 16.06-31.45] vs 9.74 [95% CI, 8.99-10.55]) and vehicular crashes (incidence rate per 1 million miles, 27.10 [95% CI, 19.95-36.80] vs 13.50 [95% CI, 12.61-14.46]) (Table 2). Analyses stratified by study site revealed that the results regarding ADHD and adverse driving outcomes were generally consistent across the sites. With adjustment for demographic and health characteristics, ADHD was associated with a 7% increased risk of hard-braking events in older drivers (aIRR, 1.07; 95% CI, 1.02-1.12), a 102% increased risk of self-reported traffic ticket events (aIRR, 2.02; 95% CI, 1.42-2.88), and a 74% increased risk of self-reported vehicular crashes (aIRR, 1.74; 95% CI, 1.26-2.40) (Figure).

**Table 2.** Incidence Rates of Self-Reported Traffic Ticket Events and Vehicular Crashes by Baseline Demographic and Health Characteristics, the Longitudinal Research on Aging Drivers Project

Variable	Incidence rate per 1 million miles (95% CI)	
	Traffic ticket events	Vehicular crashes
Age, y		
65-69	10.83 (9.67-12.12)	13.97 (12.66-15.43)
70-74	10.43 (9.17-11.87)	12.47 (11.08-14.04)
75-79	7.66 (6.28-9.33)	15.86 (13.82-18.20)
Sex		
Male	9.75 (8.73-10.89)	12.27 (11.12-13.55)
Female	10.35 (9.27-11.55)	15.46 (14.13-16.91)
Race and ethnicity		
Black, non-Hispanic	24.98 (20.76-30.07)	20.75 (16.93-25.42)
White, non-Hispanic	8.59 (7.85-9.41)	13.10 (12.18-14.10)
Other^a^	13.48 (10.22-17.79)	16.45 (12.80-21.14)
Marital status		
Married	8.34 (7.51-9.27)	12.27 (11.25-13.38)
Not married	13.46 (11.97-15.14)	17.13 (15.43-19.00)
Education		
High school or less	7.36 (5.51-9.82)	14.39 (11.71-17.69)
Associate’s degree	8.23 (6.93-9.78)	12.22 (10.61-14.07)
Bachelor’s degree	9.77 (8.29-11.52)	13.28 (11.53-15.29)
Advanced degree	11.86 (10.61-13.26)	15.01 (13.60-16.58)
Annual household income, $		
≤49 999	12.07 (10.42-13.99)	16.85 (14.87-19.09)
50 000-99 999	9.16 (8.06-10.40)	13.07 (11.74-14.54)
≥100 000	9.98 (8.70-11.46)	12.70 (11.24-14.35)
Urbanicity		
Urban	13.25 (12.19-14.40)	15.73 (14.57-16.99)
Suburban or rural	3.80 (3.06-4.72)	10.13 (8.87-11.58)
ADHD		
Yes	22.47 (16.06-31.45)	27.10 (19.95-36.80)
No	9.74 (8.99-10.55)	13.50 (12.61-14.46)
Anxiety		
Yes	13.63 (11.01-16.88)	20.44 (17.17-24.35)
No	9.68 (8.90-10.52)	13.13 (12.22-14.11)
Depression		
Yes	14.70 (12.60-17.16)	20.09 (17.60-22.93)
No	9.07 (8.28-9.93)	12.52 (11.60-13.52)
No. of medications		
0-4	9.69 (8.38-11.19)	12.64 (11.14-14.34)
5-9	9.38 (8.26-10.65)	13.22 (11.87-14.71)
≥10	11.18 (9.74-12.85)	15.83 (14.09-17.78)

Abbreviation: ADHD, attention-deficit/hyperactivity disorder.

^a^ Other race and ethnicity includes Asian, Native and Indigenous American, and Hispanic participants.

**Figure.**
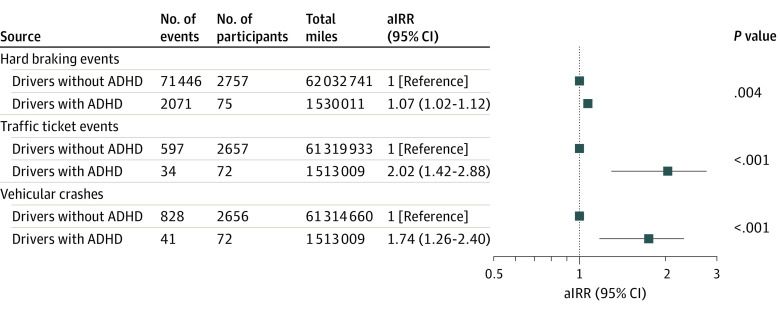
Adjusted Incidence Rate Ratios (aIRRs) of Hard-Braking Events, Self-Reported Traffic Ticket Events, and Self-Reported Vehicular Crashes Associated With Attention-Deficit/Hyperactivity Disorder (ADHD), the Longitudinal Research on Aging Drivers Project The estimated aIRRs of hard-braking events were adjusted for marital status, annual household income, urbanicity, depression, and number of medications; estimated aIRRs of traffic ticket events were adjusted for marital status, urbanicity, and depression; and estimated aIRRs of vehicular crashes were adjusted for sex, annual household income, urbanicity, anxiety, and depression.

## Discussion

The results of this prospective cohort study indicate that the prevalence of ADHD in US older adult drivers between July 2015 and March 2017 was 2.6%, compatible to the prevalence reported in the Netherlands (2.8%) and Sweden (3.3%).^25,26^ Furthermore, we found that ADHD was associated with increased risks of hard-braking events and self-reported traffic ticket events and vehicular crashes among older adult drivers. Extending prior research in adolescent and young adult drivers,^16,17,18,19^ our findings show that older adults with ADHD may experience an elevated risk of adverse driving outcomes compared with their counterparts without ADHD. Our findings are consistent with previous studies showing that ADHD is associated with an increased risk of vehicular crashes in adolescents and young adults.^14,15,27,28,29,30,31^

Many studies have examined the influences of demographic characteristics and medical conditions on the prevalence of ADHD.^2,25,28,29,30,31^ In alignment with our results, older adults with ADHD did not show statistically significant differences in sex, income, education, or urban residence compared with their counterparts without ADHD.^25^ Our results are consistent with previous reports that anxiety and depression may be closely linked to ADHD.^8,11,12,25^ Although the prevalence of ADHD declines with advancing age,^2,25,32^ the increased risk of comorbidities, such as anxiety and depression, could contribute to reduced life expectancy in these individuals. It is noteworthy that the associations between ADHD and all 3 adverse driving safety outcomes remained significant even when adjusting for these associated disorders.

Young adults with ADHD tend to overestimate their driving ability and performance since this disorder can lead to diminished self-assessment and awareness.^14^ Older adults with ADHD might have similar issues in evaluating their own driving behaviors due to impaired executive functioning, thus leading to higher driving risks. Drivers with ADHD may have difficulty remaining alert and have a decreased reaction time, resulting in a higher likelihood of collision with obstacles.^17^ In our study, older adult drivers with ADHD had excess risks of self-reported traffic ticket events and vehicular crashes that were greater than the excess risk of hard-braking events. These differences could be partly due to the limited validity and reliability of hard-braking events as a proxy measure for crashes.^33,34,35,36^

Our study also showed a threshold effect of the level of polypharmacy on the risk of adverse driving outcomes. Nonstimulant pharmacologic treatments for ADHD, such as atomoxetine, have been shown to be effective in improving symptoms as well as reducing driving errors,^37,38^ but it is unclear whether reducing polypharmacy use might have beneficial effects on driving performance. Some stimulant pharmacologic medications, such as methylphenidate,^14,39,40^ have been found to improve ADHD symptoms and help alleviate driving errors compared with placebo. A recent study, however, found increased odds of vehicular crashes for individuals treated with stimulants.^41^ Research supports that although drivers with ADHD who do not take medication may experience more inattention and impatience than drivers with ADHD who take medication, those taking methylphenidate committed a similar number of driving errors as those without ADHD or a history of taking relevant stimulant medications during on-road driving tests.^42^

Issues of underdiagnosis and treatment of ADHD in older adults still exist.^10^ Challenges of diagnoses include late emergence of obvious symptoms in adulthood and recall bias of childhood-onset symptoms in older adults.^43^ Late-life cognitive disorders, such as dementia, could obfuscate the diagnosis of ADHD due to shared pathogenic features.^10,44^ Meanwhile, the current *Diagnostic and Statistical Manual of Mental Disorders* (Fifth Edition) criteria^45^ for ADHD only specify the threshold or the number of applicable symptoms for diagnosis in children, adolescents, and adults, without giving special consideration to older adults. Given its association with driving safety alone, ADHD in older adults merits greater attention.

### Limitations

This study has several limitations. First, ADHD status was based solely on self-report, which may make the results susceptible to recall bias. Moreover, data on current ADHD symptoms and severity were not available. Second, the LongROAD project participants were not a nationally representative sample since they were disproportionately non-Hispanic White with relatively high levels of educational attainment and household income. Therefore, our findings may not be generalizable to the US older adult driver population. Third, despite being widely used in naturalistic driving studies,^33,34,35,36^ hard-braking events and self-reported traffic ticket events as proxies for unsafe driving behaviors limit the ability to draw causal inferences between ADHD and crash risk. Finally, our results are based on observational data and could be somewhat biased by unmeasured confounders, such as weather and road conditions.

## Conclusions

The results of this cohort study indicate that the lifetime prevalence of ADHD in older adult drivers was 2.6%. Older adult drivers with ADHD may be at elevated risks of hard-braking events, traffic ticket events, and vehicular crashes. As aging of the driver population continues, effective interventions to improve the diagnosis and clinical management of ADHD among older adults are warranted to promote safe mobility and healthy aging.

## Notes

Supplement.
Data Sharing Statement
