# Supplementary material for: Motor Vehicle Crash Risk in Older Adult Drivers With Attention-Deficit/Hyperactivity Disorder
Source: JAMA Netw Open. 2023 Oct 4;6(10):e2336960. doi: 10.1001/jamanetworkopen.2023.36960 (PMC10551766; doi:10.1001/jamanetworkopen.2023.36960)
Supplement: Supplement. — Data Sharing Statement [file jamanetwopen-e2336960-s001.pdf]

Data Sharing Statement

Liu. Motor Vehicle Crash Risk in Older Adult Drivers With Attention-Deficit/Hyperactivity Disorder. *JAMA Netw Open*. Published October 04, 2023.
doi:10.1001/jamanetworkopen.2023.36960

Data
Data available: No

Additional Information
Explanation for why data not available: Restrictions apply to the availability of these data. Data may be available from the author with permission from the AAA Foundation for Traffic Safety and upon execution of a data use agreement, with limitations on use.
